# Assessing Older Adults’ Decision-Making Capacity for Independent Living: Practice Tensions and Complexities

**DOI:** 10.1177/07334648211065029

**Published:** 2022-01-21

**Authors:** Ruth Usher, Tadhg Stapleton

**Affiliations:** 1 Department of Occupational Therapy and Occupational Science, 37437 University College Cork, Ireland; 2 Discipline of Occupational Therapy, 8809 Trinity College Dublin, Ireland

**Keywords:** decision-making capacity, independent living, older adults, occupational therapy, focus groups

## Abstract

Decision-making capacity (DMC) is a salient issue due to increasing ageing populations and associated dementia-related diseases. Legislative and policy developments emphasise older adults’ rights to participate in decision-making. Fifty-two occupational therapists working with older adults from a range of practice settings in Ireland participated in focus groups to discuss their contribution to multidisciplinary assessments of older adult’s DMC for independent living. Findings indicate lack of shared understanding of DMC and conflicting philosophies of practice and highlight the need for a comprehensive and multidisciplinary approach. Findings also highlight that older people are often excluded from care-planning, and independent living options are determined by availability of community services rather than their preferences. Future research will attempt to inform practice in assessing and supporting older adults’ DMC for independent living.

## Introduction

Decision-making capacity (DMC) refers to an individual’s is ability to understand and remember information relevant to a particular decision and use this information to make and communicate a choice (Barry & Docherty, 2018). It is generally assumed that adults have DMC for everyday choices and decisions with more serious consequences, regarding healthcare, finances and where to live. However, with ageing population trends and associated prevalence of chronic conditions that may impact on decision-making, methods to assess DMC among older adults are increasingly required (Bremault-Phillips et al., 2018; Charles et al., 2017; Moye & Marson, 2007). There is a need for healthcare professionals (HCPs) working with older adults to accurately and reliably assess DMC (Pennington et al., 2018) and to promote person-centred outcomes that are least restrictive (Brémault-Phillips et al., 2018).

**Figure 1.**
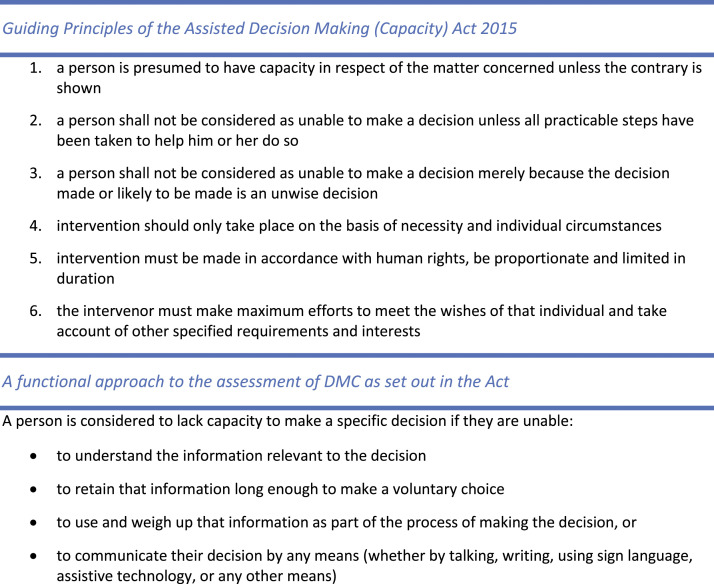
Guiding principles and functional approach to decision-making capacity (DMC) assessment.

Internationally, legislative and policy developments have drawn attention to how older adults’ DMC is assessed and supported. In many western countries, DMC-related legislation has been developed to align with the United Nations Convention of the Rights of Person with Disabilities (UNCRPD) (United Nations, 2006) and the underpinning assumption that adults are competent in decision-making. However, approaches to determining incapacity differ across jurisdictions according to variance in legal standards. In Ireland, the Assisted Decision Making (Capacity) Act 2015 provides the legal framework and definitions of DMC, emphasising that DMC is time, issue and context specific. While many states in the US determine incapacity based on combined criteria of a disabling condition, functional behaviour and cognitive functioning, Irish legislation does not require a diagnostic component as a causal condition to initiate DMC assessment. Additionally, Irish legislation does not consider a ‘best interest’ approach for substitute decision-making but places the ‘will and preference’ of individuals at the centre of all decision-making, as emphasised in UNCRPD. The guiding principles underpinning the Irish legislation and the approach to DMC assessment are set out in Figure 1.

Traditionally the treating physician, psychiatrist or psychologist assessed a person’s DMC, and this clinical judgement may have been informed by input from the multidisciplinary team (MDT). However, recent legislation and literature has emphasised the role of all HCPs in determining a person’s DMC, and Irish legislation states that the DMC assessment be carried out by the HCP with expertise specific to the area of DMC under question. Recent national and international literature refers to nurses, occupational therapists, physicians, physiotherapists, psychiatrists, psychologists, social workers and speech and language therapists being involved in DMC assessments, which typically occur in hospital settings (Donnelly et al., 2021; Jayes et al., 2020). However, Irish research indicates that traditional hierarchical cultures embedded within healthcare organisations may foster an approach where some HCPs’ skills and insights on DMC are overlooked (Ní She et al., 2020). Engagement of HCPs in the assessment of DMC may also be hampered by the lack of guidance documents and procedures for HCPs to apply the legislative requirements of DMC assessment in practice. Research highlights that many HCPs find DMC assessment challenging, noting inadequate knowledge of the concepts of DMC or how to undertake DMC assessment, competing demands within pressurised healthcare settings and lack of MDT collaboration as factors that impede HCPs engagement in DMC assessment (Davies et al., 2019; Donnelly et al., 2021; Ganzini et al., 2003; Jayes et al., 2020; Lamont et al., 2017; Usher & Stapleton, 2020; Young et al., 2018).

**Figure 2.**
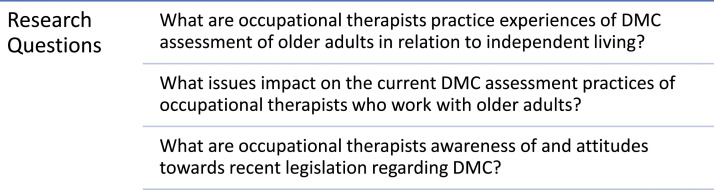
Research questions.

Independent living (IL) is one of eight DMC domains relevant to HCPs working with older adults that may require assessment (Moye & Marson, 2007). Successful functioning in IL typically includes preparing meals, shopping, managing money, medications and transportation (Lahav & Katz, 2020). It demands cognitive processes such as coping with unexpected situations and integrating multiple strategies and actions (Toglia et al., 2019). Supporting older people to live independently requires comprehensive evaluation of the older person’s health, functional capacity, resources, personal attributes, living circumstances and environment (Ahlqvist et al., 2015). Occupational therapists have a significant role in facilitating IL and it is a major part of their everyday practice with older people. While research acknowledges occupational therapists’ involvement in DMC assessment relating to discharge destination and IL decisions (Emmett et al., 2013; Jayes et al., 2020; Scott et al., 2020), limited research exploring the role of occupational therapy in DMC assessment has been conducted to date. Occupational therapists must explore the potential of their roles in assessing and supporting DMC, to ensure that individuals receive the required support to maximise their participation in decision-making about important aspects of their lives, such as IL. This study aimed to explore the role of occupational therapy in the assessment of older adults’ DMC in relation to IL, within an Irish practice context (Figure 2).

## Design and Methods

Focus groups are effective in assessing attitudes, opinions and experiences relative to a specific context (Green, 2019) and were used to explore the experiences of occupational therapists undertaking DMC assessment of older adults. An interpretive descriptive methodology (Thorne, 2016) was used to examine the subjective experience of participants and generate knowledge that could inform clinical practice. Ethical approval was granted by the university’s School of Medicine Research Ethics Committee (reference number: 20190304) prior to commencement of data collection. Purposive sampling was used to recruit practicing occupational therapists with experience of contributing to DMC assessment of older adults. The Association of Occupational Therapists of Ireland distributed study information via email to its membership. Written informed consent was obtained from individuals prior to their participation on the study. Demographic information regarding each participant’s background and experience was gathered.

The first author conducted eight focus groups over a two-month period in 2019. The focus groups employed open-ended questions following a topic guide which was informed by the literature and was adapted over the course of data collection to account for emerging findings (Additional file). Focus groups lasted 60–120 min and were digitally recorded and transcribed verbatim. Data collection continued until saturation was achieved, when comments and patterns began to repeat and little new material was generated. Nvivo 12 was used to manage the data, which was thematically analysed (Braun et al., 2018). Data familiarisation was achieved by listening to recordings and reading the transcripts to gain an overview of the breadth of content. Preliminary codes were produced by the first author and discussed and compared by both authors. Additional codes were identified and examined in relation to each other and sorted into preliminary themes. A narrative for each theme was written and themes were reviewed to ensure each theme was distinct.

Field notes and a reflective log were recorded by the first author and peer debriefing between authors was conducted to discuss ambiguous statements and development of themes. In addition to within-interview member checking, participant validation was sought through a synthesised member-checking process (Birt et al., 2016). Participants were sent preliminary interpretations of the data and invited to provide further perspectives and feedback on the provisional themes.

## Findings

### Participant Profile

Fifty-two occupational therapists, practicing across seven counties in the Republic of Ireland participated in the focus group discussions. Socio-demographic and professional profiles are provided in Table 1. Participants worked across a range of hospital and community settings, including primary care and private practice. Participants had an average of 9.5 years of experience, with the majority of participants having 7 years or more experience (57.69%, *n* = 30).

**Table 1.** Focus Group Participant Characteristics.

Demographic profile	Overall sample (n)	%
Total number of participants	52	100
Female	51	98.08
Current position grade		
Staff grade	22	42.31
Senior grade	25	48.08
Clinical specialist	1	1.92
Manager	4	7.69
Years of work experience		
Less than 3 years	10	19.23
4–6 years	12	23.08
7–10 years	12	23.08
11–15 years	9	17.31
16–20 years	3	5.77
More than 20 years	6	11.54
Mean years’ experience (standard deviation)	9.5 (*SD* 6.6708)	
Highest level of education completed		
Diploma	1	1.92
BSc	28	53.85
PG Certificate	1	1.92
PG Diploma	1	1.92
MSc	20	38.46
PhD	1	1.92
Predominant client group currently work with		
Older adults	43	82.69
Persons with stroke	6	11.54
Persons with dementia	1	1.92
Persons with developmental/intellectual disabilities	2	3.85
Practice setting		
Hospital-based	31	59.62
Community-based	20	38.46
Private practice	1	1.92
Geographical location/county currently work in		
Dublin	39	75
Louth	6	11.54
Mayo	1	1.92
Kerry	1	1.92
Meath	1	1.92
Westmeath	3	5.77
Longford	1	1.92

**Figure 3.**
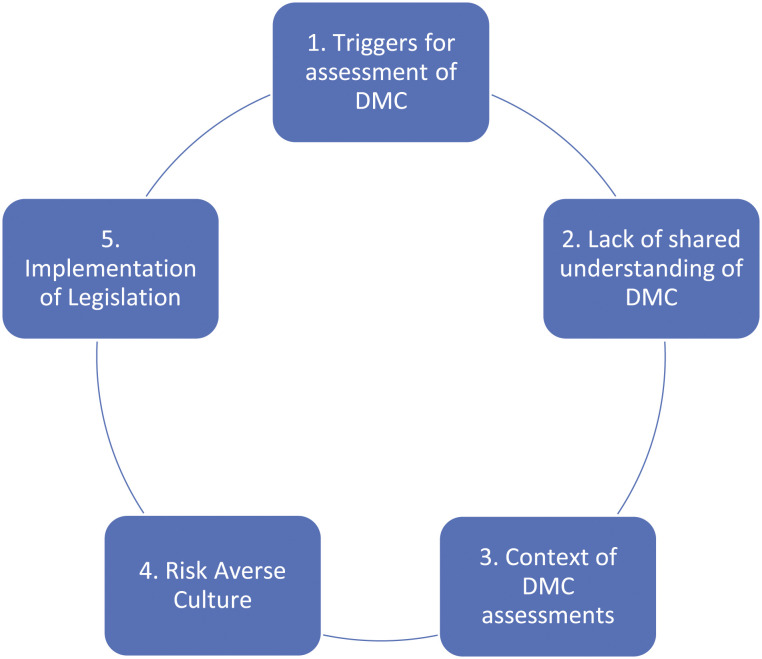
Emergent themes.

Occupational therapists in hospital and community settings reported involvement in DMC assessment of older adults regarding continuing to live at home, transitioning to residential care and acceptance of recommended equipment or support services. Therapists described the complexities and challenges of implementing DMC assessment in their practice, and areas of tension between the stakeholders involved. Five themes were identified (Figure 3) which are presented with reference to the Guiding Principles from recent DMC legislation.

#### Theme 1: Triggers for Decision-Making Capacity Assessment

While participants broadly understood the fundamental tenet that DMC of the older person is to be presumed (as per Guiding Principle 1, Figure 1), a range of triggers for assessment of DMC for IL were identified, such as when an older person presented with cognitive impairment, reduced insight to their functional abilities or support needs or concerns regarding self-neglect. Overall, indication for DMC assessment arose due to concerns for the older person’s safety, whether this risk was observed behaviour or reported by family members or other HCPs. DMC assessment was often initiated when there was a discrepancy between the views of the older person and their family, and/or the recommendations of HCPs. If the older person complied with the proposed plan for their care, it was generally assumed they had DMC, rather than completing any formal assessment. However, if the older person’s preferred options for IL were not supported by their family or HCPs, the older person’s DMC was typically questioned and assessed.

It’s when there’s a conflict between what the patient wants and what the family want…The family say ‘Oh, they can’t go home’ and ‘Go to long-term care’. If the patient doesn’t question it, generally they go to long-term care, but if they stand up and say ‘Actually, I don’t want to’, it starts to become more of an issue. FG7P3

Given the magnitude of risk involved in IL decisions, the tendency to interpret disagreement between the older person’s wishes and recommendations of HCPs and/or family as evidence of impaired DMC was further compounded when cognitive impairment was present.

They’re nearly automatically the one that’s making the ‘wrong’ or the ‘unwise’ decision and the assumption is the family are making the ‘good’ decision. FG6P4

Participants clarified that making ‘unwise’ choices did not mean the person did not have DMC (Guiding Principle 3), yet these concerns often trigger MDT discussion about the person’s DMC rather than further exploration to clarify the older person’s values and choices (Guiding Principles 4 and 6). Practice tensions occur because these triggers tend to outweigh the presumption of capacity (Guiding Principle 1).

#### Theme 2: Understanding of Decision-Making Capacity

Lack of common understanding of DMC among the MDT leads to variance in practice between different organisations, within services, and even within teams. Participants reported that not all MDT members had undertaken DMC training and were not familiar with key concepts underpinning legislation, such as the presumption of DMC and onus on the HCP to maximise and support the person’s DMC. Some HCPs treat DMC as a global, all-or-nothing trait, rather than time- and issue-specific, raising concern that the outcome of one DMC assessment might be applied to all future situations.

People talk about capacity like it’s this big global concept, you’ve got it or you don’t. Not ‘Do you have the capacity to decide you want to go home?’, ‘Do you have the capacity to decide you want to go to rehab?’… I don’t even know that I’ve had a client that has been deemed to have capacity for one element and not another FG4P3

Invariably, participants reported that among the MDT, DMC is often conflated with cognition. The terms ‘cognition’ and ‘capacity’ are used interchangeably, impacting on the assessment approach and perpetuating the misunderstanding that those with cognitive impairment do not have DMC. Participants described trying to shift the MDT focus away from inappropriate use or over-reliance on cognitive screening by emphasising functional performance assessments to supplement and contextualise information.

They’re like ‘Oh, a poor score in the cognitive assessment, that means they don’t have capacity, done’. Whereas at least if we’re involved, we can say ‘No, they scored poorly on assessment but their functional cognition is higher than that, here’s what they’re actually doing’. FG4P6

Participants also discussed how there is often a lack of clear distinction between the concepts of assessing *functional capacity* for IL or assessing *capacity to make a decision* about IL. Participants recognised the interrelatedness of these issues but emphasised understanding the distinct aspects of these concepts to ensure assessment focuses on DMC.

Maybe they can’t manage their medication, but they can decide who they want to help them manage their medication… that kind of subtle difference that’s there, they physically can’t do a task but they still have the actual capacity to decide how. FG5P2

#### Theme 3: Context of Decision-Making Capacity Assessments

Participants reported that most DMC assessments for IL occur in hospital settings. Participants highlighted the constraints of these settings, such as staffing shortages, time pressures, and resource issues, including lack of opportunity to complete home visits, as negatively impacting on their practice. Participants reported the significance and complexities of IL decisions are not always appreciated and institutional administrative pressures to discharge and maintain ‘patient flow’ often demand an efficient decision-making process, at the expense of a thorough person-centred approach.

They are not even given time or privacy to make a decision. The conversations are held at bedsides with the curtain pulled, other patients around, people listening, people running in and out of the ward, for a decision about where you’re going to go…that has massive consequences…. FG5P3

Participants felt DMC assessments would be better in the person’s own environment where the ‘*balance of power is in their favour’* (FG1P3), rather than the hospital setting, where the power imbalance typically favours the HCP and/or family. Participants described incidents of coercion and deception by HCPs and family members:

I’ve often seen them really try to coerce the person, ‘You’ll go to a nursing home, you’ll be safe there, there’s lots to do there’… Or telling them, ‘You’re only going for a few days to the nursing home, it’s not forever’…It’s a bare faced lie, that’s very uncomfortable, terrible. FG4P6

Such practice made participants feel uneasy and demonstrates that the person’s will and preference is not always considered, and furthermore the older person is excluded from the decision-making process.

Nobody asked the patient questions, nobody asked about where they wanted to go. As a rule our patients weren’t invited into the family meetings. FG7P4

Some participants attributed this to underlying ageism, which undervalues the view of the older person. Others viewed it as professional arrogance, whereby HCPs assert their expertise without due consideration for the older person:

A little bit of ‘we know best, we’re the professionals and we know what’s best for you… and a nursing home is for you’. FG4P3

Sometimes this position of authority is granted by the older person who is willing to take advice of HCPs, even if it is not what they wanted. Participants acknowledged the complexity of situations when older adults’ IL preferences are dependent on the support of family members:

We don’t necessarily have one client… it’s their own decision to make but they’re living with family members and it’s affecting other people… there’s one decision to be made and two different perspectives and it’s not necessarily clear whose decision it is to make. FG6P2

Participants reported the lack of variety and availability of community and home-based services to enable older adults continued IL complicates DMC assessment for IL:

We would very often see people who go into nursing homes before they need to because the package of care isn’t there for them in the community–‘We can’t give you 7-hours of care a week in the community, so here, have 168-hours in a nursing home instead’. FG7P2

Participants recognised that if adequate services were available to support continued community living, DMC assessment in hospital settings when planning discharge might be a less contentious issue:

Capacity wouldn’t be as much of a question because there wouldn’t be as many risks. It shouldn’t be that the services available are determining the capacity assessment FG4P3

In the absence of community services, participants recognised that family members provide care or funding to facilitate the older person’s IL preferences. Therefore, the family’s concerns cannot be precluded. Nonetheless, participants expressed frustration at the dominance of the family’s voice over the patient’s will and preference:

They’ve way too much power. The family’s preference takes precedence over the patient preference. If they’re willing to support the risky discharge, they go in your favour. But if they’re not, even if the discharge isn’t that risky, but they don’t fill the gaps in carers, then it’s just not a reality. FG1P8

#### Theme 4: Risk Averse Culture

Participants described the dominant focus within healthcare as adherence to risk management which is at odds with person-centred DMC assessment as outlined in legislation, and also in conflict with their professional philosophy. Participants described the culture of risk aversion and subsequent restrictive practices as representing ‘*a total conflict of interest with your professional values’* (FG3P5).

HCPs’ recommendations often adhered to the institution’s risk-minimisation focus, subsequently compromising the older person’s choice and autonomy, and potentially denying rights and opportunities to engage in valued occupations.

We’ve gotten so caught up in risk that we negate human rights. FG1P7.

Participants described a reluctance among MDTs to support positive risk-taking to enable continued IL and participation in valued occupations due to a narrow understanding of risk among the MDT. Physical risk is easier to quantify and measure, therefore there is a tendency to address issues such as falls risk. Conversely, the risk posed to the older adult’s well-being by denying them opportunities to make choices or to engage in valued occupations is rarely considered and this is at odds with occupational therapy’s philosophy.

We predominantly focus on physical risk anyway and we don’t really look at psychological risk to somebody’s health and wellbeing, to their overall occupational identity… We don’t have any equation for psychological risk and that long-term effect for people, of not being able to fulfil and live their lives the way they want do, even with an impairment or a disability. FG6P3

HCPs are also compromised by threats of litigation from families who were not satisfied to support the older person’s IL preferences. Fear of professional repercussions causes HCPs to become more concerned with protecting themselves by choosing restrictive practices, rather than considering the consequences for the person.

It’s very risk averse here. When they’re going home, there’s nearly always a panic and it’s all the outcome for ourselves, rather than the patient. Like the whole litigation thing, people are really vulnerable with it. FG5P1

#### Theme 5: Implementation of Legislation

Recent legislation has increased awareness among occupational therapists of the need to support older people to make their own decisions about IL. However, the lag between the enactment and implementation of the legislation caused confusion and frustration. Lack of guidance on implementation has led to reluctance to engage in DMC assessment and this is further hindered by competing policies and priorities. Participants described difficulty reconciling various policy directives which seem contradict each other, such as a focus on errorless practice which does little to support IL decision-making:

As a manager, all I hear in the healthcare environment is quality improvement, patient safety, errorless practice. What can we do to reduce errors? We’re meant to be supporting people in terms of making decisions…it is a real challenge from legislation. FG8P3

## Discussion

This study aimed to explore occupational therapists’ practices in assessment of older adults’ DMC for IL. Findings regarding the triggers and challenges of DMC assessment were consistent with contemporary literature on DMC. This discussion considers the findings which enhance our understanding of the tensions HCPs experience in reconciling the need to respect older people’s decisions and views and address the risks relating to their choices.

A key trigger for DMC assessment identified in this study is discrepancy between the older person’s choice and the family member and/or HCPs recommendations. When the older person does not agree with family member’s safety concerns or a HCP’s recommendations, they are construed as lacking insight. Echoing previous studies, these findings suggest that when an older person’s IL decision concurs with family and/or HCP recommendations, DMC is not doubted (Capron, 2015; Ganzini et al., 2003). Wade (2019) cautions that if it is wrong to assume lack of DMC on the basis of an unwise decision, it is wrong to assume DMC because the decision agreed is what the HCPs thinks is best. Therefore, IL decisions, such as place of residence, require attention from HCPs who should be satisfied the person has DMC or the required supports to make that decision, in keeping with the of the legislation.

Cognitive impairment is associated with difficulties performing daily activities (Toth et al., 2021), and thus is a significant consideration in DMC assessment for IL. Occupational therapists argued that cognitive-function is best understood within the context of performing activities of daily living, as older people often compensate for deficits by utilising other skills or aids. However, they reported that HCPs over-rely on cognitive screens to inform DMC recommendations. There is growing criticism that standard cognitive assessments lack ecological validity and do not adequately address the dynamic interaction between cognitive, motor and social skills, activity demands and activity context (Ossher et al., 2013; Romero-Ayuso et al., 2021). Existing approaches to cognitive-functional evaluation incorporate patient report, collateral report, occupation-based assessment and cognitive screening, allowing occupational therapists to make clinical judgements about function, cognition and safety (Erez & Katz, 2018; Giles et al., 2020) and these should be considered in DMC assessments for IL.

Similar to previous recommendations, occupational therapists supported a DMC assessment approach that includes detailed clinical interview, assessments of cognition, function, social and environmental status and mood, along with values and preferences of the person (Brindles and Holmes, 2005; Moberg & Rick, 2008; Sinclair et al., 2019). However, exclusion of some older people from decision-making processes is a significant finding from this study requiring urgent change in HCPs’ practices. Recent DMC discourse focuses on promoting autonomy, thus allowing older people make their own decisions. However, participants reported that families and professionals dominate the older person’s IL decisions, confirming other research findings that older people are denied opportunity to partake in decision-making (Donnelly et al., 2019; Durocher et al., 2017; Sinclair et al., 2019).

Relational autonomy offers practical insights to engaging older adults and their families in decision-making, by recognising the social and political contexts in which decisions are made. This emphasizes multi-directional information sharing, whereby HCPs learn about the person’s life circumstances, needs, values and preferences and the person learns of the various options available to them, allowing a decision to be reached that aligns with the person’s wishes (Durocher et al., 2017; Sherwin & Winsby, 2011). However, occupational therapists in this study described paternalistic approaches where consideration for the older adult’s physical safety outweighed any other aspect of wellbeing. Ryff and Keyes (1995) propose that psychological well-being encompasses other important dimensions such as autonomy, environmental mastery and personal growth. Promoting autonomy requires recognising risk as an integral part of life, yet while HCPs report a commitment to upholding autonomy, they only do so if the clients’ preference is deemed to be safe (Durocher et al., 2017). Participants highlighted the need to go beyond measuring and minimising proximal and physical risk and to consider broader implications of psychological risk on wellbeing and quality of life. The predominant focus on safety, compounded by lack of services, may explain why HCPs privileged their own expertise over the perspectives of the older adults in decision-making. However, lack of shared understanding of DMC and the implications of recent legislation among the MDT and family, along with lack of resources and fear of litigation, raise many practice issues.

While HCPs are motivated to engage in DMC assessment and to support older people’s participation in decision-making processes, several barriers impede their practice such as such as time pressures, staffing issues and lack of clarity in professional roles, similar to previous findings (Charles et al., 2017; Davies et al., 2019; Jayes et al., 2020; Sinclair et al., 2019). This study highlights the need to improve fragmented and inequitable access to community care, and how few alternatives to nursing homes exist for older adults who are unable to continue living independently, as has been acknowledged in literature (Carter et al., 2019; Walsh et al., 2015). Resource limitations, systemic constraints and differing team values preclude ethical decision-making that is consistent with professional values in occupational therapy practice (Bushby et al., 2015; Hazelwood et al., 2019; VanderKaay et al., 2019). Hammell (2007) argues that occupational therapists often actively reinforce and perpetrate restrictive organisational policies that disempower people and challenges the profession to demonstrate accountability to the client rather than the organisation. Senior leadership, sufficient resources and policies are required to implement optimal assisted decision-making practices (Davies et al., 2019). In order to implement legislation rooted in human rights and needs-led approaches, healthcare services must review their structures and processes and HCPs, such as occupational therapists, who claim a commitment to person-centred practice must reflect on their practices.

Findings indicate a lack of shared understanding of DMC among HCPs in Ireland, thus education and training to promote a thorough understanding of DMC concepts, principles and legislative requirements is necessary. Previous research suggests education should include legal and ethical content, communication skills training, mentorship, and specifically address the needs of people with dementia (Davies et al., 2019; Ní Shé et al., 2020). DMC education and training for HCPs should promote collaborative, interdisciplinary working and emphasize that Irish legislation does not prescribe which HCPs should assess DMC. Similar to other international studies (Borrett & Gould, 2020; Manthorpe & Samsi, 2016; Murrell & McCalla, 2016), family members of older people may not understand how DMC legislation affects their relatives’ lives. Therefore, initiatives to raise public awareness of the implications of DMC legislation in Ireland may enable older people and their family members to understand their rights, roles and responsibilities.

## Limitations

A limitation of this study is that it relates to the Irish context, though participants represented a wide range of practice settings across a wide geographical area. Findings may be transferable to many occupational therapists in Ireland, and potentially internationally; however, the legislative, policy and funding environment should be considered in observations drawn. As DMC assessment for IL is a complex area that requires input from multiple stakeholders, perspectives from other HCPs working with older people may provide valuable insights that would enhance interprofessional collaboration in this area. Lastly, research from the perspectives of older people, and other relevant stakeholders such as family members may further elaborate on the findings from this study (Reid & Reid, 2005).

## Conclusion

Findings illustrate many tensions and complexities within existing DMC assessment practices in relation to IL for older adults. Practice constraints and resource issues are highlighted, along with issues relating to supporting IL decisions which may encompass risk-taking. As findings to date highlight a gap between awareness of DMC legislation and its subsequent application into everyday practice, subsequent research will aim to facilitate consensus on procedures for occupational therapists in Ireland to address DMC assessment for IL of older adults, from a client-centred, occupation-based perspective that aligns with legislative changes.

With increasing societal interest in the subject of DMC and evolving appreciation of autonomy and rights, insights offered on DMC practice issues are much needed. Understanding the issues involved in assessing and supporting older people to participate in decision-making for IL is an important concern for all HCPs who contribute to these processes.

## Supplemental Material

sj-pdf-1-jag-10.1177_07334648211065029 – Supplemental Material for Assessing Older Adults’ Decision-Making Capacity for Independent Living: Practice Tensions and Complexities Click here for additional data file. Supplemental Material, sj-pdf-1-jag-10.1177_07334648211065029 for Assessing Older Adults’ Decision-Making Capacity for Independent Living: Practice Tensions and Complexities by Ruth Usher, and Tadhg Stapleton in Journal of Applied Gerontology

## References

[bibr1-07334648211065029] Ahlqvist A. Nyfors H. Suhonen R. (2016). Factors associated with older people's independent living from the viewpoint of health and functional capacity: A register-based study. Nursing Open, 3(2), 79–89. 10.1002/nop2.39 27708818 PMC5047332

[bibr2-07334648211065029] Barry C. Docherty M. (2018). Assessment of mental capacity and decision-making. Medicine, 46(7), 405–410. https://doi-org.ucc.idm.oclc.org/10.1016/j.mpmed.2018.0

[bibr3-07334648211065029] Birt L. Scott S. Cavers D. Campbell C. Walter F. (2016). Member checking: A tool to enhance trustworthiness or merely a nod to validation? Qualitative Health Research, 26(13), 1802–1811. 10.1177/1049732316654870. 27340178

[bibr44-07334648211065029] Borrett S. Gould L. J. (2020) Mental capacity assessment with people with aphasia: understanding the role of the speech and language therapist, *Aphasiology*, 10.1080/02687038.2020.1819954

[bibr4-07334648211065029] Braun V. Clarke V. Terry G. Hayfield N. (2018). Thematic analysis. In Liamputtong P. (Ed.), Handbook of Research methods in health and social sciences (pp. 843-860). Springer.

[bibr5-07334648211065029] Brémault-Phillips S. Pike A. Charles L. Roduta-Roberts M. Mitra A. Friesen S. Moulton L. Parmar J. (2018). Facilitating implementation of the decision-making capacity assessment (DMCA) model: Senior leadership perspectives on the use of the national implementation research network (NIRN) model and frameworks. BMC Research, 11(1), 607. 10.1186/s13104-018-3714-x PMC6107947 30139366

[bibr6-07334648211065029] Brindle N. Holmes J. (2005). Capacity and coercion: Dilemmas in the discharge of older people with dementia from general hospital settings. Age and Ageing, 34(1), 16–20. 10.1093/ageing/afh228 15496463

[bibr7-07334648211065029] Bushby K. Chan J. Druif S. Ho K. Kinsella E. A. (2015). Ethical tensions in occupational therapy practice: A scoping review. British Journal of Occupational Therapy, 78(4), 212–221. 10.1177/0308022614564770

[bibr45-07334648211065029] Capron A. M. (2015). Not Taking “Yes” for an Answer. The Journal of clinical ethics, 26(2), 104–107. 26132056

[bibr8-07334648211065029] Carter L. O’Neill S. Keogh F. Pierce M. O’Shea E. (2019). Intensive home care supports, informal care and private provision for people with dementia in Ireland. Dementia 10.1177/1471301219863580 31349753

[bibr9-07334648211065029] Charles L. Parmar J. Brémault-Phillips S. Dobbs B. Sacrey L. Sluggett B. (2017). Physician education on decision-making capacity assessment: Current state and future directions. Canadian Family Physician, 63(1), e21–e30. 10.1093/geroni/igx004.769. 28115457 PMC5257236

[bibr10-07334648211065029] Davies C. Fattori F. O'Donnell D. Donnelly S. Ní Shé É. O Shea M. Prihodova L. Gleeson C. Flynn Á. Rock B. Grogan J. O'Brien M. O'Hanlon S. Cooney M. T. Tighe M. Kroll T. (2019). What are the mechanisms that support healthcare professionals to adopt assisted decision-making practice? A rapid realist review. BMC Health Services Research, 19(1), 960. 10.1186/s12913-019-4802-x 31831003 PMC6909502

[bibr11-07334648211065029] Donnelly S. Begley E. O'Brien M. (2019). How are people with dementia involved in care-planning and decision-making? An Irish social work perspective. Dementia, 18(7–8), 2985–3003. 10.1177/1471301218763180 29544346

[bibr12-07334648211065029] Donnelly S. Ó Coimín D. O'Donnell D. Ní Shé É. Davies C. Christophers L. Mc Donald S. Kroll T. (2021). Assisted decision-making and interprofessional collaboration in the care of older people: a qualitative study exploring perceptions of barriers and facilitators in the acute hospital setting. Journal of Interprofessional Care, 35(6), 852–862. 10.1080/13561820.2020.1863342 33588668

[bibr13-07334648211065029] Durocher E. Gibson B. E. Rappolt S. (2017). Mediators of marginalisation in discharge planning with older adults. Ageing and Society, 37(9), 1747–1769. 10.1017/S0144686X16000593

[bibr14-07334648211065029] Emmett C. Poole M. Bond J. Hughes J. C. (2013). Homeward bound or bound for a home? Assessing the capacity of dementia patients to make decisions about hospital discharge: comparing practice with legal standards. International Journal of Law and Psychiatry, 36(1), 73–82. 10.1016/j.ijlp.2012.11.009 23187119

[bibr15-07334648211065029] Erez A. B. Katz N. (2018). Cognitive functional evaluation. In Katz N. Toglia J. (Eds.), Cognition, occupation, and participation across the life span: Neuroscience, neurorehabilitation, and models of intervention in occupational therapy. AOTA Press.

[bibr16-07334648211065029] Ganzini L. Volicer L. Nelson W. Derse A. (2003). Pitfalls in assessment of decision-making capacity. Psychosomatics, 44(3), 237–243. 10.1176/appi.psy.44.3.237 12724505

[bibr17-07334648211065029] Giles G. M. Edwards D. F. Baum C. Furniss J. Skidmore E. Wolf T. Leland N. E. (2020). Making functional cognition a professional priority. American Journal of Occupational Therapy, 74(1), 7401090010p1. 10.5014/ajot.2020.741002 PMC7018454 32078504

[bibr18-07334648211065029] Green J. (2019). The use of focus groups in health research. In Saks M. Allsop J. (Eds.), Researching health: Qualitative, quantitative and mixed methods. SAGE Publications Ltd.

[bibr19-07334648211065029] Hammell K. W. (2007). Client-centred practice: ethical obligation or professional obfuscation? British Journal of Occupational Therapy, 70(6), 264–266. 10.1177/030802260707000607

[bibr20-07334648211065029] Hazelwood T. Baker A. Murray C.M. Stanley M. (2019). New graduate occupational therapists’ narratives of ethical tensions encountered in practice. Australian Journal of Occupational Therapy, 66(3), 283–291. 10.1111/1440-1630.12549 30548269

[bibr21-07334648211065029] Jayes M. Palmer R. Enderby P. Sutton A. (2020). How do health and social care professionals in England and Wales assess mental capacity? A literature review. Disability and Rehabilitation, 42(19), 2797–2808. 10.1080/09638288.2019.1572793 30739505

[bibr22-07334648211065029] Lahav O. Katz N. (2020). Independent older adult’s IADL and executive function according to cognitive performance. OTJR: Occupation, Participation and Health, 40(3), 183–189. 10.1177/1539449220905813 32107963

[bibr23-07334648211065029] Lamont S. Stewart C. Chiarella M. (2017). Capacity and consent: Knowledge and practice of legal and healthcare standards. Nursing Ethics, 26(1), 71–83. 10.1177/0969733016687162 28093938

[bibr46-07334648211065029] Manthorpe J. Samsi K. (2016). Care homes and the Mental Capacity Act 2005: Changes in understanding and practice over time. Dementia, 15(4), 858–871. 10.1177/1471301214542623. 25015949

[bibr24-07334648211065029] Moberg P. J. Rick J. H. (2008). Decision-making capacity and competency in the elderly: a clinical and neuropsychological perspective. Neuro Rehabilitation, 23(5), 403–413. 10.3233/nre-2008-23504 18957727

[bibr25-07334648211065029] Moye J. Marson D. C. (2007). Assessment of decision-making capacity in older adults: An emerging area of practice and research. Journals of Gerontology-Series B Psychological Sciences and Social Sciences, 62(1), P3–P11. 10.1093/geronb/62.1.P3 17284555

[bibr47-07334648211065029] Murrell A McCalla L (2016). Assessing Decision-making Capacity: The Interpretation and Implementation of the Mental Capacity Act 2005 Amongst Social Care Professionals. Practice, 28(1), 21–36. 10.1080/09503153.2015.1074667.

[bibr26-07334648211065029] Ní Shé E. O’Donnell É.D. Donnelly S. Davies C. Fattori F. Kroll T. (2020). “What bothers me most is the disparity between the choices that people have or don’t have”: A qualitative study on the health systems responsiveness to implementing the assisted decision-making (capacity) act in Ireland. International Journal Environment Research and Public Health, 17(9), 32–94. 10.3390/ijerph17093294 PMC7246817 32397345

[bibr27-07334648211065029] Ossher L. Flegal K. E. Lustig C. (2013). Everyday memory errors in older adults. Neuropsychology, development, and cognition. Section B, Aging, Neuropsychology and Cognition, 20(2), 220–242. 10.1080/13825585.2012.690365 PMC3443516 22694275

[bibr28-07334648211065029] Pennington C. Davey K. ter Meulen R. Coulthard E. Kehoe P. G. (2018). Tools for testing decision-making capacity in dementia. Age and Ageing, 47(6), 778–784. 10.1093/ageing/afy096 30010696

[bibr29-07334648211065029] Reid D. J. Reid F. J. M. (2005). Online focus groups: An in-depth comparison of computer-mediated and conventional focus group discussions. International Journal of Market Research, 47(2), 131–162. 10.1177/147078530504700204

[bibr30-07334648211065029] Romero-Ayuso D. Castillero-Perea Á. González P. Navarro E. Molina-Massó J. P. Funes M. J. Ariza-Vega P. Toledano-González A. Triviño-Juárez J. M. (2021). Assessment of cognitive instrumental activities of daily living: a systematic review. Disability and Rehabilitation, 43(10), 1342–1358. 10.1080/09638288.2019.1665720 31549907

[bibr31-07334648211065029] Ryff CD Keyes CL (1995). The structure of psychological well-being revisited. Journal of Personality and Social Psychology, 69(4), 719–727. 10.1037/0022-3514.69.4.719 7473027

[bibr32-07334648211065029] Scott J. Weatherhead S. Daker-White G. Manthorpe J. Mawson M. (2020). Practitioners’ experiences of the mental capacity act: a systematic review. The Journal of Adult Protection, 22(4), 227–244. 10.1108/JAP-02-2020-0005

[bibr33-07334648211065029] Sherwin S. Winsby M. (2011). A relational perspective on autonomy for older adults residing in nursing homes. Health Expectations, 14(2), 182–190. 10.1111/j.1369-7625.2010.00638.x 21029285 PMC5060573

[bibr34-07334648211065029] Sinclair C. Bajic-Smith J. Gresham M. Blake M. Bucks R. S. Field S. Kurrle S. (2019). Professionals’ views and experiences in supporting decision-making involvement for people living with dementia. Dementia. 10.1177/1471301219864849 31349752

[bibr35-07334648211065029] Thorne S. (2016). Interpretive description qualitative research for applied practice (2nd ed.). Routledge.

[bibr36-07334648211065029] Toglia J. P. Gilisz K.M. Goverover Y. (2019). Cognition, perception and occupational performance. In Schell B. A. B. Gilen G. (Eds.), Willard and Spackman’s occupational therapy (13th ed., pp. 901–941). Wolters Kluwer Health.

[bibr37-07334648211065029] Toth C. Tulliani N. Bissett M. Liu K. P. (2021). The relationship between cognitive function and performance in instrumental activities of daily living in older adults. British Journal of Occupational Therapy. 10.1177/03080226211008722. PMC12033700 40336786

[bibr38-07334648211065029] United Nations (2006). Convention on the rights of persons with disabilities. The United Nations. 10.1515/9783110208856.203 18348362

[bibr39-07334648211065029] Usher R. Stapleton T. (2020). Occupational therapy and decision‐making capacity assessment: A survey of practice in Ireland. Australian Occupational Therapy Journal, 67(2), 110–120. 10.1111/1440-1630.12629 31769037

[bibr40-07334648211065029] VanderKaay S. Jung B. Letts L. Moll S. E. (2019). Continuing competency in ethical decision making: An interpretive description of occupational therapists’ perspectives. Canadian Journal of Occupational Therapy, 86(3), 209–219. 10.1177/0008417419833842 31092004

[bibr41-07334648211065029] Wade D. T (2019). Determining whether someone has mental capacity to make a decision: clinical guidance based on a review of the evidence. Clinical Rehabilitation, 33(10), 1561–1570. 10.1177/0269215519853013 31169035

[bibr42-07334648211065029] Walsh K. Carney G.M. Leime A.N. (2015). Ageing through austerity: Critical perspectives from Ireland. Policy Press.

[bibr43-07334648211065029] Young G. Douglass A. Davison L. (2018). What do doctors know about assessing decision-making capacity? New Zealand Medical Journal, 131(1471), 58–71. 29518800

