# Supplementary material for: Assessing Older Adults’ Decision-Making Capacity for Independent Living: Practice Tensions and Complexities
Source: J Appl Gerontol. 2022 Jan 21;41(5):1264–73. doi: 10.1177/07334648211065029 (PMC9024017; doi:10.1177/07334648211065029)
Supplement: sj-pdf-1-jag-10.1177_07334648211065029 – Supplemental Material for Assessing Older Adults’ Decision-Making Capacity for Independent Living: Practice Tensions and Complexities [file sj-pdf-1-jag-10.1177_07334648211065029.pdf]

Additional file: Focus Group Topic Guide

DMC assessment process

- *What triggers DMC assessment?*
- *What is your role in the DMC assessment process?*
- *How do you assess DMC?*
- *What information needs to be considered?*
- *Who needs to be involved? (patients, families/carers, MDT professionals, advocates)*
- *Any particular assessment tools that you are aware of/ that are useful?*
- *How do you support patients during an assessment?*

DMC attitudes and beliefs

- *How do you find DMC assessment? What are the major gaps and barriers in the capacity assessment process as its being done now, and what are its effects?*
- *How confident do you feel in your level of DMC assessment expertise (knowledge and skills)? In what areas would you like further information and/or training?*
- *What current resources do you rely on to help support you in DMC assessment? What other resources or facilitators do you think would help in your role in DMC assessment?*

DMC legislation

- *Please describe your experience with DMC assessment related legislation or legal issues*
- *Has it influenced any changes in your practice (knowledge, skills and attitudes)?*
- *Have you experienced any barriers to implementing legislation in practice? If so, how have you overcome these barriers? If not, why not?*
- *Do you feel confident working with this legislation*
